# Natural menstrual rhythm and oral contraception diversely affect exhaled breath compositions

**DOI:** 10.1038/s41598-018-29221-z

**Published:** 2018-07-18

**Authors:** Pritam Sukul, Jochen K. Schubert, Phillip Trefz, Wolfram Miekisch

**Affiliations:** Rostock Medical Breath Research Analytics and Technologies (ROMBAT), Dept. of Anesthesiology and Intensive Care, University Medicine Rostock, Schillingallee 35, D-18057 Rostock, Germany

## Abstract

Natural menstrual cycle and/or oral contraception diversely affect women metabolites. Longitudinal metabolic profiling under constant experimental conditions is thereby realistic to understand such effects. Thus, we investigated volatile organic compounds (VOCs) exhalation throughout menstrual cycles in 24 young and healthy women with- and without oral contraception. Exhaled VOCs were identified and quantified in trace concentrations via high-resolution real-time mass-spectrometry, starting from a menstruation and then repeated follow-up with six intervals including the next bleeding. Repeated measurements within biologically comparable groups were employed under optimized measurement setup. We observed pronounced and substance specific changes in exhaled VOC concentrations throughout all cycles with low intra-individual variations. Certain blood-borne volatiles changed significantly during follicular and luteal phases. Most prominent changes in endogenous VOCs were observed at the ovulation phase with respect to initial menstruation. Here, the absolute median abundances of alveolar ammonia, acetone, isoprene and dimethyl sulphide changed significantly (*P-*value ≤ 0.005) by 18.22↓, 13.41↓, 18.02↑ and 9.40↓%, respectively. These VOCs behaved in contrast under the presence of combined oral contraception; e.g. isoprene decreased significantly by 30.25↓%. All changes returned to initial range once the second bleeding phase was repeated. Changes in exogenous benzene, isopropanol, limonene etc. and smoking related furan, acetonitrile and orally originated hydrogen sulphide were rather nonspecific and mainly exposure dependent. Our observations could apprehend a number of known/pre-investigated metabolic effects induced by monthly endocrine regulations. Potential *in vivo* origins (e.g. metabolic processes) of VOCs are crucial to realize such effects. Despite ubiquitous confounders, we demonstrated the true strength of volatolomics for metabolic monitoring of menstrual cycle and contraceptives. These outcomes may warrant further studies in this direction to enhance our fundamental and clinical understanding on menstrual metabolomics and endocrinology. Counter-effects of contraception can be deployed for future noninvasive assessment of birth control pills. Our findings could be translated toward metabolomics of pregnancy, menopause and post-menopausal complications via breath analysis.

## Introduction

The natural endocrine regulation in young and healthy adult women during their menstrual cycle is an everlasting clinical interest^1,2^. Monthly sinus physiological and endocrine changes affect both metabolism and cellular biochemistry^3^ in menstrual women. Moreover, daily ingested oral contraception^4^ has obvious counter-effects and hindrance to the natural monthly rhythm of sex hormones in adults. Due to diverse physiological and/or therapeutic effects, follow-up of different phases of menstrual cycle^5,6^ is challenging^7,8^. Longitudinal *in vivo* assessment of metabolites throughout the entire menstrual cycle can enlarge our knowledge on the metabolic status of women. Metabolic changes during the follicular- and luteal phases and especially at ovulation are of special interest^9^ and may extend our conventional understanding of female endocrinology and effects of synthetic hormone therapy (i.e. via oral contraception).

Noninvasive analysis of exhaled breath volatile organic compounds (VOCs) is a steadily evolving domain in metabolomics^10–12^. Application of volatile metabolomics via repeated measurements on comparable biological cohorts within a consistent state-of the-art experimental model may avoid many confounders^13–15^ and variabilities^16^.

VOCs are either produced endogenously or absorbed and stored from habits and/or environment^17^. Thus, breath volatiles inherit potential for noninvasive assessment of physiology^18,19^, pathophysiology^20–22^ and therapy^23–25^. Thousands of volatile metabolites have already been quantified in trace concentrations (~ppbV–pptV range) in our exhalation. Human physiology plays the crucial role in bronco-pulmonary gas-exchange and thereby, on VOC exhalation^26,27^. Thus, real-time profiling of exhaled alveolar VOC concentrations can contribute new insight into biochemical- and metabolic changes^28,29^ induced by normal or abnormal physiological effects. Real-time mass-spectrometric (MS) techniques e.g. proton transfer reaction (PTR)-Time of flight (ToF)-MS^30,31^ along with end-tidal/alveolar sampling^32,33^ have enabled online monitoring of immediate physiological changes within split-seconds^34–36^. These studies showed that concentration changes may be more important than the presence of unique breath VOC biomarkers.

In this perspective, effects of metabolic changes during normal menstrual cycle or effects induced by oral contraceptive pills are the prime concern of this study. Here, we follow-up changes in exhaled VOC concentrations from young and healthy women during their normal menstrual cycles and also throughout the cycles undergoing combined oral contraception by applying high-resolution PTR-ToF-MS. The following questions are addressed herein:

- What are the effects of natural menstrual cycle onto exhaled breath VOC concentrations in young and healthy women?
- Do those effects differ under combined oral contraceptive medication?
- Are these changes related to known/pre-investigated metabolic effects induced by endocrine regulations throughout the cycle with- or without contraception?
- Does the ovulation phase effect VOC concentrations in women without contraception?

## Results

We observed pronounced differences in VOC concentrations throughout the menstrual cycle. These changes followed the female sex hormone regulation. Different behaviors were clearly noticeable between both groups of women. In both cohorts, these changes were VOC specific. Observed changes in endogenous and blood-borne VOCs were assignable to the ovulation phase in the cohort without contraception (from here on referred to as “control cohort”). Most VOC concentrations returned to initial levels once the reference phase (menstrual bleeding) was repeated in both cohorts.

**Figure 1:**
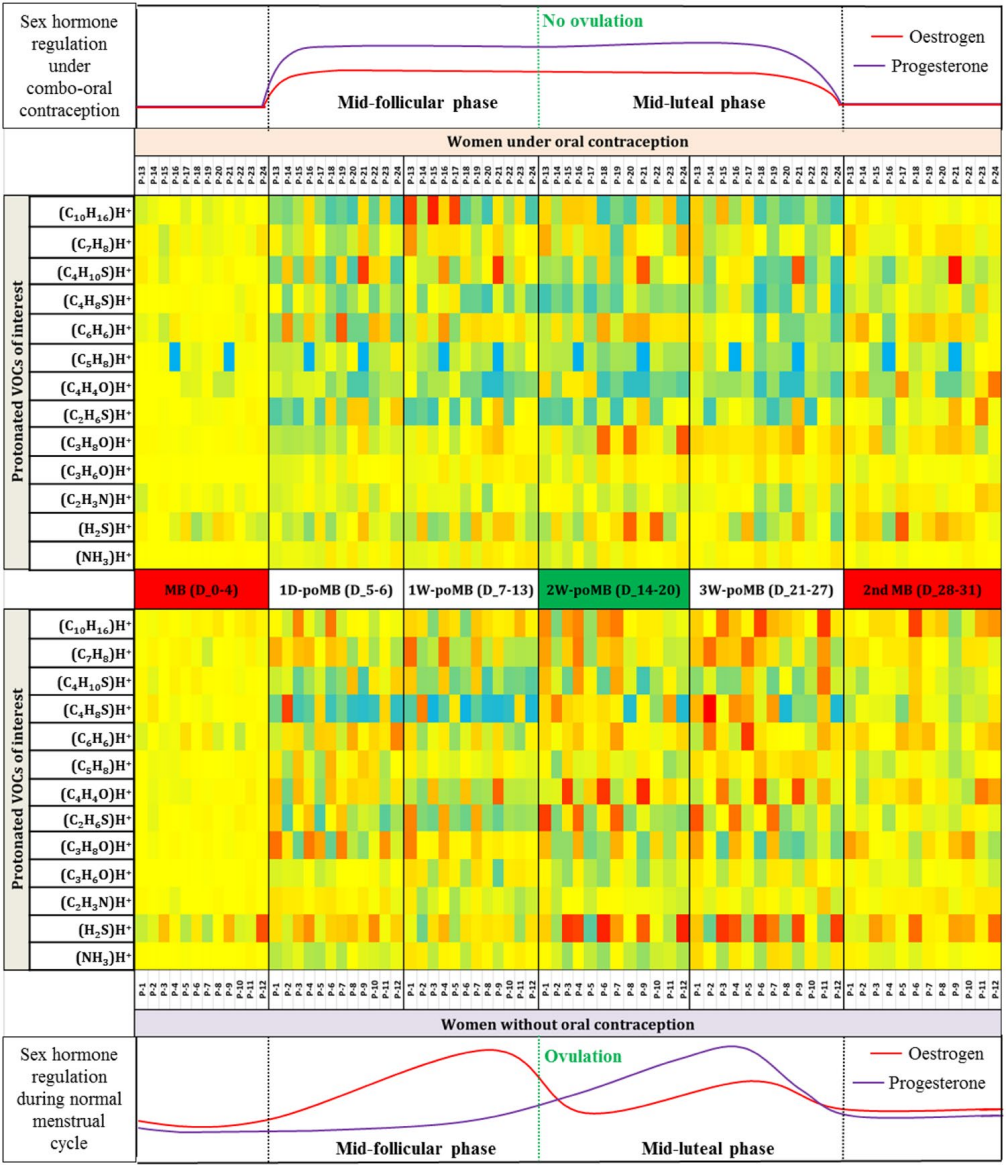
Heat-map of exhaled end-tidal abundances of 13 selected VOCs in 24 young and healthy women throughout six different phases of the menstrual cycle (menstrual bleeding – 2^nd^ menstrual bleeding). Medians of normalized values over 60 s of breathing are displayed for each participant during the different cycle phases (located as the central X-axis). VOC data were normalized (for each participant) onto corresponding abundances in the third breath of the second minute from the initial menstrual bleeding phase (see method section). Red, green and blue colors represent relatively high, medium and low concentrations, respectively. The upper heat-map represents the data from the contraception cohort (P-13–P-24) and the lower heat-map represents the data from the control group (P-1–P-12). Isoprene was not exhaled by P-16 and P-21 (contraception cohort) and was thus assigned to ‘0’ values in all measurement points. Qualitative regulations of two main female sex hormones are placed at the top (of contraception cohort) and bottom (of control cohort) along the x-axis. Red and violet lines represent estrogen and progesterone hormones, respectively. The vertical green line represents the onset of the ovulation phase and separates the follicular and luteal phases.

Figure 1 represents a semi-quantative expression of relative changes for selected breath VOC markers over the course of the entire study within the two cohorts of women (with- and without oral contraceptives). Normalized median values from each participant over 60 s of breathing are displayed. The 13 VOCs included in the heat-map were selected as they had significantly higher concentration in expiration than in inspiratory air. Inspiratory concentrations did not change during the measurements.

### Changes in absolute abundances of VOCs during different phases of the menstrual cycle

Every indicated changes refer to the initial menstrual bleeding phase (MB). In the following section, only the most important and significant (i.e. *P*-value ≤ 0.005) changes are presented with a numeric value (in %) and all other cases are listed in Table 1 or Supplementary Table S1.

- *Changes on the day after the bleeding stopped:*
  *Overall changes*: Median values of exhaled alveolar ammonia (NH_3_) decreased by 2.3%. Dimethyl sulphide (C_2_H_6_S, DMS), allyl-methyl-sulphid (C_4_H_8_S, AMS) and limonene (C_10_H_16_) concentrations decreased by 48.8, 26.7 and 5.2%, respectively.
  *Changes in control cohort*: Ammonia decreased by 17.0%. Acetonitrile (C_2_H_3_N) increased by 21.6%.
  *Changes in contraception cohort*: DMS, AMS and isopropanol concentrations decreased by 26.7, 15.5 and 28.0%, respectively.
- *Changes during the mid-follicular phase:*
  *Overall changes*: Acetone (C_2_H_6_O) increased by 6.4%. DMS, AMS and acetonitrile decreased by 64.5, 56.6 and 10.5%, respectively.
  *Changes in control cohort*: AMS decreased by 71.9%.
  *Changes in contraception cohort*: DMS and AMS decreased by 36.8 and 39.5% respectively.
- *Changes during the ovulation phase:*
  *Overall changes*: Ammonia, acetone and isoprene (C_5_H_8_) dropped by 9.9, 11.0 and 19.5%, respectively. Benzene (C_6_H_6_) increased by 19.1%. DMS reached 51.8% below the initial range.
  *Changes in control cohort*: Ammonia and acetone dropped by 18.2 and 13.4%, respectively. Isoprene increased by 18.0%.
  *Changes in contraception cohort*: Isoprene decreased by 30.2%. DMS and AMS also decreased by 47.0 and 53.4%, respectively.
- *Changes during the mid-luteal phase:*
  *Overall changes*: Acetone increased by 11.9%. DMS reached 46.8% below the initial baseline.
  *Changes in control cohort*: Isoprene also increased by 48.8%. Limonene increased by 60.0%. Isopropanol decreased by 12.3%.
  *Changes in contraception cohort*: DMS and AMS reached 35.6 and 38.5%, respectively below the initial level. Isopropanol increased by 35.5%.
- *Changes during 2nd menstrual phase:*

**Table 1:** Statistical significance of observed changes in exhaled abundances of eight different VOCs in each cohort. Median values of VOC concentrations from the second minute (/60 s) of every measurement points were compared.

Protonated VOCs [g/mol]	Control cohort		Phases of menstrual cycle	Contraception cohort		Control cohort		Phases of menstrual cycle	Contraception cohort		Protonated VOCs [g/mol]
	Significance (*P* ≤ 0.005)/Median/60 s	Median Changes (%)/60 s		Median Changes (%)/60 s	Significance (*P* ≤ 0.005)/Median/60 s	Significance (*P* ≤ 0.005)/Median/60 s	Median Changes (%)/60 s		Median Changes (%)/60 s	Significance (*P* ≤ 0.005)/ Median/60 s	
(Ammonia)H + 18.03382	***N/A***	**0**	***MB***	**0**	***N/A***	***N/A***	**0**	***MB***	**0**	***N/A***	(Isopropanol)H + 61.06479
	*0.004*	−17.09	**1D-poMB**	3.32	>0.00*5*	>0.00*5*	38.74	**1D-poMB**	−27.99	<0.00*1*	
	>*0.005*	4.50	**1W-poMB**	8.35	>*0.005*	>*0.005*	8.89	**1W-poMB**	3.04	>*0.005*	
	<*0.001*	−18.22	**2W-poMB**	3.83	>*0.005*	>*0.005*	7.92	**2W-poMB**	18.61	>*0.005*	
	>*0.005*	12.56	**3W-poMB**	9.00	>*0.005*	*0.001*	−12.34	**3W-poMB**	35.54	*0.002*	
	>*0.005*	0.43	**2nd MB**	0.76	>*0.005*	>*0.005*	18.04	**2nd MB**	19.99	>*0.005*	
(Acetone)H + 59.04914	***N/A***	***0***	***MB***	***0***	***N/A***	***N/A***	***0***	***MB***	***0***	***N/A***	(AMS)H + 89.04195
	>*0.005*	−10.48	**1D-poMB**	−2.02	>*0.005*	>*0.005*	−61.10	**1D-poMB**	−15.55	<*0.001*	
	>*0.005*	11.34	**1W-poMB**	5.75	>*0.005*	<*0.001*	−71.89	**1W-poMB**	−39.48	*0.005*	
	<*0.001*	−13.41	**2W-poMB**	8.62	>*0.005*	>*0.005*	−39.47	**2W-poMB**	−53.38	<*0.001*	
	>*0.005*	5.37	**3W-poMB**	15.97	>*0.005*	>*0.005*	−10.40	**3W-poMB**	−38.54	*0.002*	
	>*0.005*	−2.96	**2nd MB**	−2.09	>*0.005*	>*0.005*	−8.61	**2nd MB**	−16.07	*0.001*	
(Isoprene)H + 69.06989	***N/A***	***0***	***MB***	***0***	***N/A***	***N/A***	***0***	***MB***	***0***	***N/A***	(Limonene)H + 137.13248
	>*0.005*	42.83	**1D-poMB**	−23.31	>*0.005*	>*0.005*	−4.04	**1D-poMB**	−13.76	>*0.005*	
	>*0.005*	2.91	**1W-poMB**	−28.31	>*0.005*	>*0.005*	46.92	**1W-poMB**	−2.00	>*0.005*	
	*0.003*	18.02	**2W-poMB**	−30.25	*0.005*	>*0.005*	54.82	**2W-poMB**	10.92	>*0.005*	
	<*0.001*	48.77	**3W-poMB**	−17.88	>*0.005*	<*0.001*	59.96	**3W-poMB**	−21.43	>*0.005*	
	>*0.005*	−11.87	**2nd MB**	−3.87	>*0.005*	>*0.005*	25.44	**2nd MB**	19.04	>*0.005*	
(DMS)H + 63.0263	***N/A***	***0***	***MB***	***0***	***N/A***	***N/A***	***0***	***MB***	***0***	***N/A***	(Acetonitrile)H + 42.03382
	>*0.005*	−44.05	**1D-poMB**	−26.71	<*0.001*	*0.003*	21.64	**1D-poMB**	−5.11	>*0.005*	
	>*0.005*	−50.11	**1W-poMB**	−36.78	<*0.001*	>*0.005*	−19.38	**1W-poMB**	−5.50	>*0.005*	
	>*0.005*	−9.40	**2W-poMB**	−47.09	*0.003*	>*0.005*	−9.08	**2W-poMB**	0.47	>*0.005*	
	>*0.005*	−7.37	**3W-poMB**	−35.59	*0.001*	>*0.005*	−22.77	**3W-poMB**	−5.59	>*0.005*	
	>*0.005*	−3.82	**2nd MB**	19.63	*0.001*	<*0.001*	15.01	**2nd MB**	26.83	<*0.001*	

Median Changes (%)/60 s: positive values represent an increase and negative values represent a decrease in in comparison to the initial MB phase. Significance (*P*-value ≤ 0.005)/Median/60 s: statistically significant differences between the reference value (from MB phase) and actual values from all other measurement points were assessed by means of repeated measurement-ANOVA on ranks. Changes with a resulting p-value ≤ 0.005 were considered as significant.

*Overall changes*: DMS, AMS and acetonitrile remained significantly different to initial baseline.

*Changes in control cohort*: Only acetonitrile did not return to initial concentrations.

*Changes in contraception cohort*: DMS, AMS and acetonitrile did not return to baseline.

Exhaled abundances of hydrogen sulphide (H_2_S), toluene (C_7_H_8_) and methyl-propyl sulphide (C_4_H_10_S) did not change significantly in any case. Surprisingly, no isoprene was exhaled by two women (P-16 and P-21) from the contraception cohort. Furan (C_4_H_4_O) was exhaled only by six smoking women from the control cohort. Although its exhalation closely mirrored isoprene no statistical relevance could be drawn due to low observation numbers. Detailed data on every observed change of different VOC concentrations and statistical significances (along with corresponding *P*-values) are listed in Table 1 and Supplementary Table S1.

### Comparisons between the six different measurement points (i.e. menstrual cycle phases)

**Figure 2:**
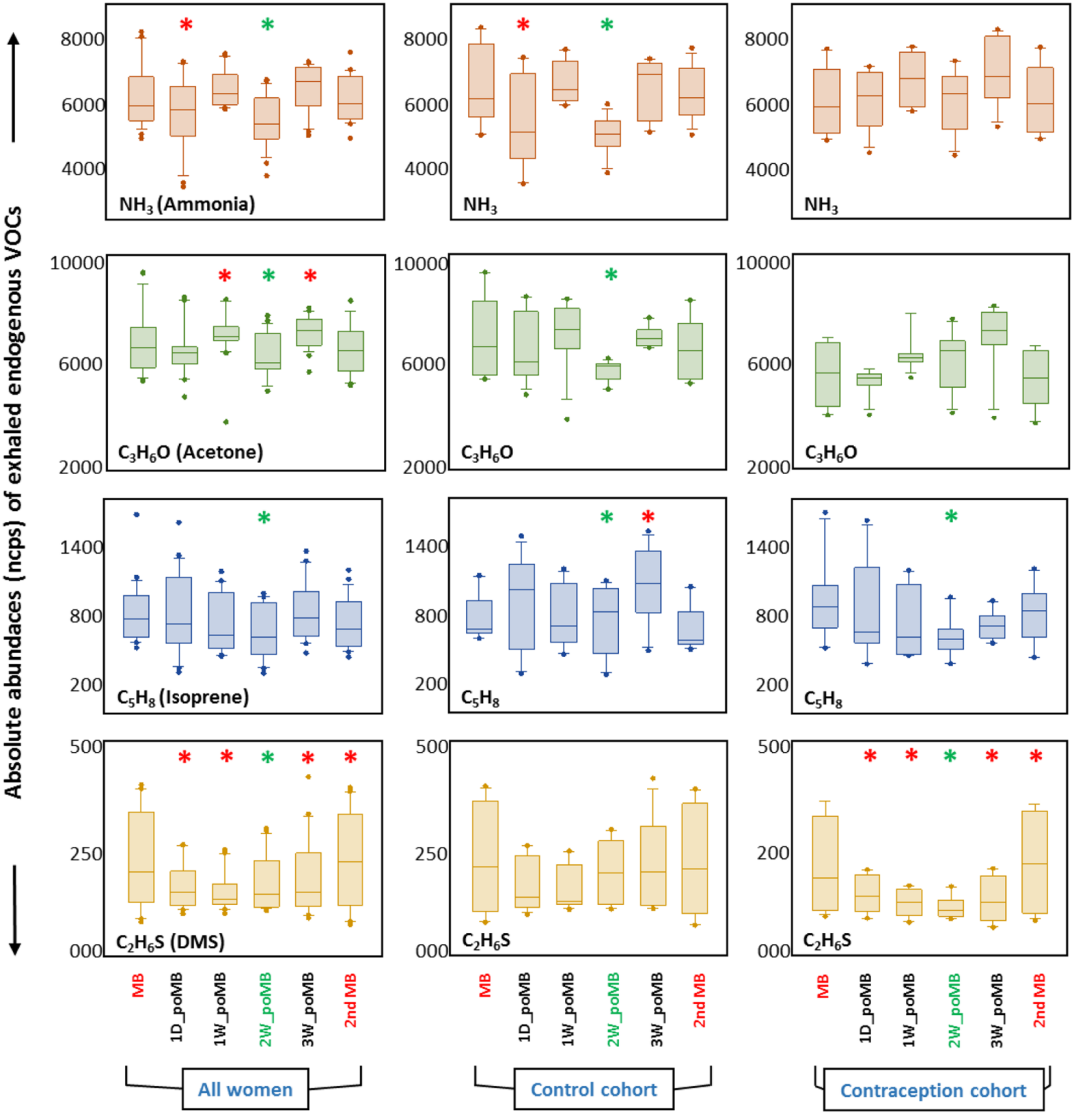
Comparisons of four endogenous VOCs from all women, control- and contraception cohort. Y-axes represent median signal intensities of exhaled endogenous VOCs. X-axes represent the six different measurement points (i.e. different menstrual cycle phases from first menstrual bleeding to 2^nd^ menstrual bleeding) corresponding to the time course of the study. VOC data of all phases were compared to the corresponding median values in the initial ‘menstrual bleeding (MB)’ phase. The ‘*’ symbols (red and green colored) represent the statistically significant (i.e. *P*-value ≤ 0.005) differences in relation to the initial MB phase. A green ‘*’ is used to assign significant changes at the ovulation phase.

Comparison of absolute median values (over 60 s) in all six different measurement points are represented as boxplots for nine selected VOCs. Four potentially endogenous VOCs from all women, control- and contraception cohort during different phases of the menstrual cycle are plotted in Fig. 2 and the same is shown for other five potentially exogenous VOCs in Supplementary Fig. S1. From all pair-wise multiple comparisons, a colored ‘*’ is assigned on those which are significantly (i.e. *P*-value ≤ 0.005) different with reference to the corresponding medians from the initial menstrual bleeding phase.

## Discussion

We observed pronounced changes in exhaled alveolar VOC concentrations throughout the menstrual cycles. These changes mirrored many investigated metabolic effects induced by monthly endocrine regulations. Eventually, VOC exhalations in the contraception cohort greatly differed compared to the control cohort. Observed changes were VOC specific. Changes in certain endogenous and blood-borne VOCs occurred at the follicular and luteal phases. These changes turned out to be most prominent at the ovulation event in the control cohort. The synthetic hormones, present in oral contraceptive pills do not appear directly in exhaled breath. Nevertheless, we could demonstrate that natural menstrual effects onto some exhaled VOCs disappeared under the presence of contraception. Our results can facilitate and inspire future investigation in noninvasive metabolic monitoring of numerous biochemical-, physiological- and metabolic processes.

### Ammonia exhalation

The major source of breath ammonia is protein metabolism^28^ and its exhalation depends on changes of blood-pH e.g. due to respiratory acidosis or alkalosis^20^. In the control cohort, significantly decreased ammonia exhalation just after periods can be attributed to menstruation driven loss of functional endometrial epithelium and consecutive low abundance of plasma progesterone. Progesterone has a catabolic effect on protein metabolism pathways^37–39^, whereas estrogen mediates the activity of cerebral and endometrial progesterone receptors^40,41^. Ammonia concentrations returned to initial range during the mid-follicular phase presumably due to an increase in number and sensitivity of progesterone receptors and elevated plasma estrogen. The most pronounced and significant decrease in exhaled ammonia concentrations occurring at the ovulation event in the control group could be assigned to the decreased estrogen and low progesterone levels. Due to the regular oral intake of these hormones by the contraception cohort, ammonia exhalation did not change significantly throughout the duration of the study.

### Acetone exhalation

Acetone is mainly produced via glucose and fat metabolism^42^. Acetone exhalation closely mirrored the ammonia profile. An overall significant increase in acetone exhalation during mid-follicular phase can be attributed to higher plasma estrogen. Estrogen and its receptors play a crucial role in cellular energy metabolism^43^. Although acute insulin action on glucose metabolism remained unaffected by estradiol treatment^44^, the risk of type-II diabetes was observed to increase with longstanding estrogen administration^45^. Estrogen stimulates blood glucose transport to cells and its metabolism by up-regulating glycolytic kinase enzymes in the cytoplasm^43^. Moreover, estrogen is accounted for improved surfactant production and alveologenesis and thereby, can increase alveolar elimination of highly water soluble compounds e.g. acetone and ammonia^46,47^. Exhaled alveolar acetone concentrations decreased significantly at ovulation in the control cohort probably due to a pronounced decline of circulating estrogen. Acetone exhalation did not behaved alike in the contraception cohort due to constant oral supply of estrogen. An overall significant rise in acetone during luteal phase can be assigned to estrogen rise. Consecutively, increased progesterone can antagonize the insulin action in adipose tissue^37^ and will increase lipolysis.

### Isoprene exhalation

Isoprene is supposed to originate predominantly from the mevalonate pathway of cholesterol biosynthesis^48^. Although liver and intestine are the central organ for total cholesterol regulation, all cells express its biosynthetic enzymes to maintain membrane integrity. Significantly elevated isoprene exhalation during ovulation phase in the control cohort can be attributed to supposedly high plasma estrogen. Estrogen and its receptor expressions facilitate mitochondrial β-oxidation of fatty acid and cytoplasmic oxidation of pyruvate, which derive Acetyl Co-A and thereby, trigger the mevalonate pathway^43^. Overall, lipoprotein cholesterol abundance changes in response to varying estrogen levels. Evidence suggests that total cholesterol and low density lipoprotein-C are maximized during the follicular phase and drop during the luteal phase, with high density lipoprotein-C highest around ovulation^49^. Cholesterol is the precursor for the synthesis of steroid hormones^50^. Significantly elevated isoprene concentrations may thus indicate an increased rate of cholesterol biosynthesis that contributes to higher plasma concentrations of both sex hormones required for the subsequent luteal phase. In human cell lines, synthetic progesterone inhibits cholesterol biosynthesis^51^. Thus, the constant intake of synthetic progesterone and estrogen may have led to a decreased isoprene concentration in the middle of the cycle in the contraception cohort. Interestingly, two participants (P-16 and P-21) who have continuously been taking combined-oral contraception for more than 10 years, exhaled no traceable isoprene. This could be due to the progesterone driven suppression of enzymes responsible for the mevalonate pathway.

### DMS exhalation

DMS is mainly produced via methylation of methionine by anaerobic gut bacterial colony^52^. Active estrogen maintains the growth and diversity of systemic flora and thereby regulates DMS exhalation. In contrast to the control cohort, a significantly decreased DMS exhalation throughout the cycles under contraception can be assigned to a possible decrease in estrogen activity. While looking for a possible cause we came across published evidences that synthetic progesterone can cause antibacterial effects on the gut flora^53^. Researchers evidenced that antibiotic administrations may often result in contraceptive failure^54,55^. As there is no certain systemic interaction between antibiotics and oral contraceptives, the explanation of such cases remained unclear. Here, the suppressed DMS exhalations in the contraception cohort indicates a possible antibacterial activity of the daily ingested synthetic progesterone via oral pills. DMS exhalation was different in the 2^nd^ menstrual bleeding phase in the contraception cohort most possibly due to the fact that the half-life of the synthetic progesterone is longer than the half-life of the natural hormone. Intestinal bacteria play an important role in estrogen metabolism by secreting β-glucuronidase, an enzyme that de-conjugates estrogen into its active form^56^. Antibiotics may cause dysbiosis of gut microbiota^57^, which can result in decreased de-conjugation and therefore, in down-regulation of plasma estrogen. Vice versa, estrogen deficiency suppresses microbial diversity and abundance of intestinal bacteria that are related to immune homeostasis^58^. Thus, our observation supports the fact that co-administration of oral contraceptive and antibiotic drugs should be avoided.

### Exhalation of exogenous VOCs

Varying and nonspecific effects were observed on exogenous VOCs. Breath hydrogen sulphide is sourced from oral bacterial emissions and AMS is known to originate from food constituent e.g. garlic, onions etc^59^. Here, hydrogen sulphide exhalation did not change whereas AMS showed similar tendencies as DMS in both cohorts. This may indicate a parallel origin of breath AMS from gut flora and its regulations via sex hormones. AMS remained different in the 2^nd^ MB phase due to similar reasons indicated for DMS exhalation. Other exogenous VOCs e.g. benzene and toluene mainly accumulate from environmental exposure^60^. The potential source of furan is smoking. Acetonitrile is related to smoking and environmental exposure. Isopropanol and limonene are used as flavoring agents in food, beverages or mouthwash as well as in disinfectants at clinical environment^59^. Due to variable inspiratory concentrations of these VOCs throughout the study period, no systematic effects of estrogen and progesterone were observed. This emphasizes the fact that determination of inspiratory concentrations is mandatory for rational clinical interpretation of exhaled breath markers.

It is well known that the natural menstrual cycle or contraceptive therapy may predominantly affect carbohydrate, glucose, lipid and protein metabolism by up- or down-regulating those pathways^37,43^. As endogenous VOCs are known to originate from or are also regulated by those metabolic processes, observed changes in VOC exhalation can be used to monitor systemic/metabolic effects induced by different menstrual phases or via oral contraception. Exogenous substances such as benzene or toluene, acetonitrile or furans may accumulate due to environmental exposition, smoking habits and intake from food or beverages. The four substances selected for monitoring (ammonia, acetone, isoprene and DMS – Table 1, Fig. 2) are known to be mainly endogenous. The fact, that characteristic changes of the selected compounds were observed during the menstrual cycle regardless of different lifestyles and nutrition further supports the hypotheses, that these changes were linked to metabolic changes rather than to lifestyle.

As our prime interest laid on the noninvasive assessment of qualitative effects due to natural menstrual changes and changes under oral contraceptives, quantitative invasive measurements of plasma hormone concentrations were not obligatory in this study. There were not any specific VOCs or volatile hormones that appeared directly in the breath of normal or contraceptive subjects. Rather than looking for specific markers, we developed an *in vivo* clinical setup to observe the actual potential of volatile profiling to follow-up natural or therapy induced metabolic changes within a biologically comparable study population. Despite vast confounding limitations and challenges in the metabolomics field, we were able to demonstrate consistent and comparable changes in all participants with substantially low variations; even in absolute values. Consequently, most of those changes were reproduced (i.e. statistically indifferent) once the reference phase was repeated in both cohorts. This result was obtained through repeated measurements, which were applied under stable, optimized and constant state-of the-art experimental conditions.

In conclusion, via applying an advanced real-time analytical technique we monitored rapidly occurring physiological and metabolic changes, induced by nature derived menstrual rhythm in young and healthy adult women. Inclusion of a biologically comparable (i.e. age, gender and BMI matched) cohort of oral contraceptive subjects allowed us to suppress the natural endocrine rhythm and reliably assign the observed changes to hormonal regulations. Observed effects were same and comparable in every participant from the same cohort. These phenomena clearly demonstrate the unique strength of noninvasive metabolic profiling of menstrual cycle homeostasis via exhaled VOC concentrations and its future possible applications for the qualitative assessment of oral contraception or hormonal therapy. These findings could expand our present state of basic and medical knowledge on menstrual endocrinology, metabolomics and clinical breath research. These outcomes are innovative, novel and significantly important to be translated into metabolic follow-up of pregnancy, menopause and post-menopausal complications (e.g. hormonal imbalance, loss of bone mineral density, osteoporosis and cardiomyopathy etc.) in ageing societies by means of noninvasive breath-gas analysis. An intelligible and comprehensive adaptation of our model into certain advanced sensor-based applications may attribute to unconventional avenues towards noninvasive point of care (PoC) monitoring of female health via exhaled breath-gas analysis in the future.

## Methods

Our experiments were carried out in accordance with Declaration of Helsinki guidelines. Ethical approval from the Institutional Ethics Committee (IEC, University Medical Centre Rostock, Germany) and signed informed consent from 24 young and healthy women (aged between 18–45 years) were obtained. Among these women, 12 were undertaking combined oral contraceptive pills (i.e. contraception cohort) and other 12 were not using any female contraception (i.e. control cohort).

Only regularly (without early or delayed period problems) menstruating women with no recent pregnancy, miscarriage or expectation were included randomly. Women with a history of any acute or chronic disease and undergoing therapy or dietary supplements were excluded. Conjugal status as well as smoking and/or drinking habits were also recorded upon inclusion. Subject’s demographic information along with clinically relevant parameters (e.g. inclusion/exclusion criteria) is listed in Table 2.

**Table 2:** Demographic information of healthy women.

Subject IDs	Age (Years)	Gender	Height (cm)	Weight (Kg)	Cigarette smoking habits	Alcohol drinking habits	BMI (Kg/m^2^)	Undertaking combined oral contraception	Irregular menstrual cycle	Recent pregnancy, miscarriage or expecting	Any acute or chronic disease	Any addiction, medication or diet	Conjugal life
1	25	F	155	48	No	No	20	No	No	No	No	No	Yes
2	45	F	168	60	Yes	No	21	No	No	No	No	No	Yes
3	28	F	168	70	Yes	No	25	No	No	No	No	No	Yes
4	20	F	163	52	No	No	20	No	No	No	No	No	Yes
5	39	F	170	67	Yes	No	23	No	No	No	No	No	Yes
6	26	F	172	69	Yes	No	23	No	No	No	No	No	Yes
7	18	F	160	55	No	No	22	No	No	No	No	No	Yes
8	35	F	166	66	No	No	24	No	No	No	No	No	Yes
9	22	F	165	71	Yes	No	26	No	No	No	No	No	Yes
10	27	F	175	63	No	No	21	No	No	No	No	No	Yes
11	41	F	163	65	Yes	No	24	No	No	No	No	No	Yes
12	19	F	169	68	No	No	24	No	No	No	No	No	Yes
13	28	F	170	63	No	No	22	Yes	No	No	No	No	Yes
14	24	F	165	71	No	No	26	Yes	No	No	No	No	Yes
15	21	F	165	64	No	No	24	Yes	No	No	No	No	Yes
16	27	F	160	55	No	No	22	Yes	No	No	No	No	Yes
17	40	F	164	70	No	No	26	Yes	No	No	No	No	Yes
18	37	F	168	75	No	No	27	Yes	No	No	No	No	Yes
19	28	F	166	68	No	No	25	Yes	No	No	No	No	Yes
20	26	F	167	71	No	No	26	Yes	No	No	No	No	Yes
21	30	F	173	77	No	No	26	Yes	No	No	No	No	Yes
22	23	F	169	60	No	No	21	Yes	No	No	No	No	Yes
23	35	F	172	71	No	No	24	Yes	No	No	No	No	Yes
24	20	F	161	63	No	No	24	Yes	No	No	No	No	Yes

Subject’s age, gender, height, body weight, smoking or drinking habits and body mass index (BMI) are listed along with additional clinically important parameters e.g. use of oral contraceptives, pregnancy, diseases or other addiction, medication or diet and conjugality etc.

### Study Protocol

We started breath sampling for each woman during her menstruation bleeding phase and then repeated follow-ups with defined intervals until the next bleeding. The series of our six measurement points were as follows:

1. Menstrual (0–4^th^ day of menstrual cycle) bleeding phase [MB (D_0–4)] →
2. 1^st^ Day (5^th^–6^th^ day of menstrual cycle) after the bleeding stops [1D-poMB (D_5–6)] →
3. 1^st^ Week after bleeding stops (i.e. mid-follicular phase) [1W-poMB (D_7–13)] →
4. 2^nd^ week (14^th^–20^th^ day of menstrual cycle) after the bleeding stops (i.e. ovulation phase) [2W-poMB (D_14–20)] →
5. 3^rd^ week (21^st^–27^th^ day of menstrual cycle) after the bleeding stops (i.e. mid-luteal phase) [3W-poMB (D_21–27)] →
6. 2^nd^ Menstrual (28^th^–31^st^ day of menstrual cycle) bleeding phase [2^nd^ MB (D_28–31)]

All volunteers rested in the sitting position for 10 min before the actual measurement in order to minimize all hemodynamic changes from preceded standing or walking. Each subject maintained a normal sitting position^18^ and performed oral breathing (inhalation and exhalation through mouth only)^59^ via a sterilized Teflon-mouthpiece of 2.5 cm diameter^61^. After performing one minute of paced breathing (with a fixed normal respiratory rate of 12 breaths/min by following the sound of a metronome) they continued another 2 min of spontaneous breathing (metronome was muted at this time). Mouthpieces were reused after sterilization. In order to avoid any partial/unsupervised nasal breathing, we used nose clips in this setup.

### PTR-ToF-MS measurements

Breath VOCs were measured continuously in real-time via a high-resolution proton transfer reaction-time of flight-mass spectrometry [PTR-ToF-MS 8000] (Ionicon Analytik GmbH, Innsbruck, Austria). The functional principle and optimized conditions of our instrument for online breath sampling and analysis were described in previous studies^26,31^. Briefly the following steps take place:

1. *PTR- Sampling mode and sample transfer*: 20 ml/min of breath are drawn continuously into the 6 m long heated (75 °C) silco-steel PTR transfer-line in side-stream mode via a t-piece connected to the sterile mouthpiece. In this way the breath sample is directly transferred into the drift tube of the instrument (please see 3).
2. *Ion source*: Hydronium ions (H_3_O^+^) are produced as primary ions from pure (99.9%) water vapor in a hollow cathode discharge ion source. These ions then then transferred to the drift tube via a lens system.
3. *Drift tube*: Sample analytes (i.e. breath VOCs) are introduced in this chamber. The soft ionizations (i.e. minimal fragmentation) of VOCs takes place via proton transfer reactions [VOC + H_3_O^+^ → (VOC)H^+^ + H_2_O]. Only VOCs with relatively higher proton affinity than water are being ionized.
4. *ToF-MS*: Protonated VOCs are then transferred from the drift tube into the high-resolution reflectron time-of-flight mass spectrometer (Tofwerk AG, Thun, Switzerland), where they get detected according to their mass to charge (m/z) ratios.

Preconcentration is not required in this technique. The following pre-optimized experimental conditions were used:

*PTR- Mass resolution*: ~4000 m/∆m (enables isobaric separation of substances).

*PTR- Time resolution*: 200 ms (i.e. after every 200 ms of interval a new mass spectrum was recorded).

*Other PTR-parameters*: 4 mA of ion source current, 6 ml/min of H_2_O flow, Drift tube temperature of 75 °C, voltage of 610 V and pressure of 2.3 mbar were used. The resulting E/N ratio was 139 Td.

*VOC data recording and mass calibration*: After every minute a new data file was recorded automatically and the mass scale was recalibrated after each run (60 s). We used the following masses for mass calibration: 21.0226 (H_3_O^+^-Isotope), 29.9980 (NO^+^) and 59.049 (C_3_H_6_O).

### VOC data processing

VOCs were measured in counts per seconds (cps) and corresponding intensities were normalized onto primary ion (H_3_O^+^) counts. As PTR measures the VOCs continuously, inspiratory/ambient and expiratory data are recorded seamlessly. Thus, we applied a custom-made data processing algorithm (‘Breath tracker’; based on MATLAB version 7.12.0.635, R2011a) to identify expiratory and inspiratory phases^31,35^. Here, we used acetone as the tracker mass due to its endogenous origin and relatively higher abundance in exhalation than in inspiratory air.

VOCs were tentatively identified via their sum formulas and protonated masses are listed in Table 1 and Table S1. The high mass resolution of the PTR-ToF-MS-8000 enables very accurate assignment of a chemical formula to its measured mass. Thus, within the discussion VOCs are referred to by their name or sum formula.

### Reduction of intra-individual variations for the heat-map

All *in vivo* studies are bound to have intra-individual variations. In this longitudinal approach, each volunteer was considered as her own control. Thus, during the presentation of relative changes throughout the menstrual cycle in the heat-map (Fig. 1), intra-individual variations were reduced by normalizing VOC concentrations onto the corresponding values in the third breath (from the second minute) of the first menstruation phase.

### Statistical analysis

Due to the non-parametric distribution of data, *median* values were considered for all statistical analysis. For statistical comparisons between the 6 different measurement points (i.e. each group) we treated the data as follows:

At first, the median (from all 24 participants) of exhaled VOC concentrations (absolute values) in each group were calculated (i.e. over the 60 s of measurement from the spontaneous breathing phase). Secondly, the same as above were calculated separately for the contraception- and control cohort (12 subjects in each cohort).

Statistically significant differences in all above-mentioned parameters were evaluated via *repeated measurement ANOVA on ranks* (Friedman repeated measures analysis of variance on ranks, Shapiro-Wilk test for normal distribution and post hoc Student–Newman–Keuls method for pairwise multiple comparisons between all groups; *P*-value ≤ 0.005) in SigmaPlot (version 13) software.

From all pairwise comparisons, we selected those referring to the corresponding values from the first menstrual bleeding phase [MB (D_0–4)].

### Data availability

Authors comply with the data availability policy of *Scientific Reports*.

## Electronic supplementary material

Supplementary Figure and Table
