# Supplementary material for: Natural menstrual rhythm and oral contraception diversely affect exhaled breath compositions
Source: Sci Rep. 2018 Jul 18;8:10838. doi: 10.1038/s41598-018-29221-z (PMC6052073; doi:10.1038/s41598-018-29221-z)
Supplement: Supplementary file 1 — Supplementary Figure and Table [file 41598_2018_29221_MOESM1_ESM.docx]

**Natural menstrual rhythm and oral contraception diversely affect exhaled breath compositions**

Pritam Sukul^1*^, Jochen K Schubert^1^, Phillip Trefz^1^, Wolfram Miekisch^1^

^1^*Rostock Medical Breath Research Analytics and Technologies (ROMBAT), Dept. of Anesthesiology and Intensive Care, University Medicine Rostock, Schillingallee 35, D-18057 Rostock, Germany*

* Corresponding author: pritam.sukul@uni-rostock.de

***Supplementary figure and table:***


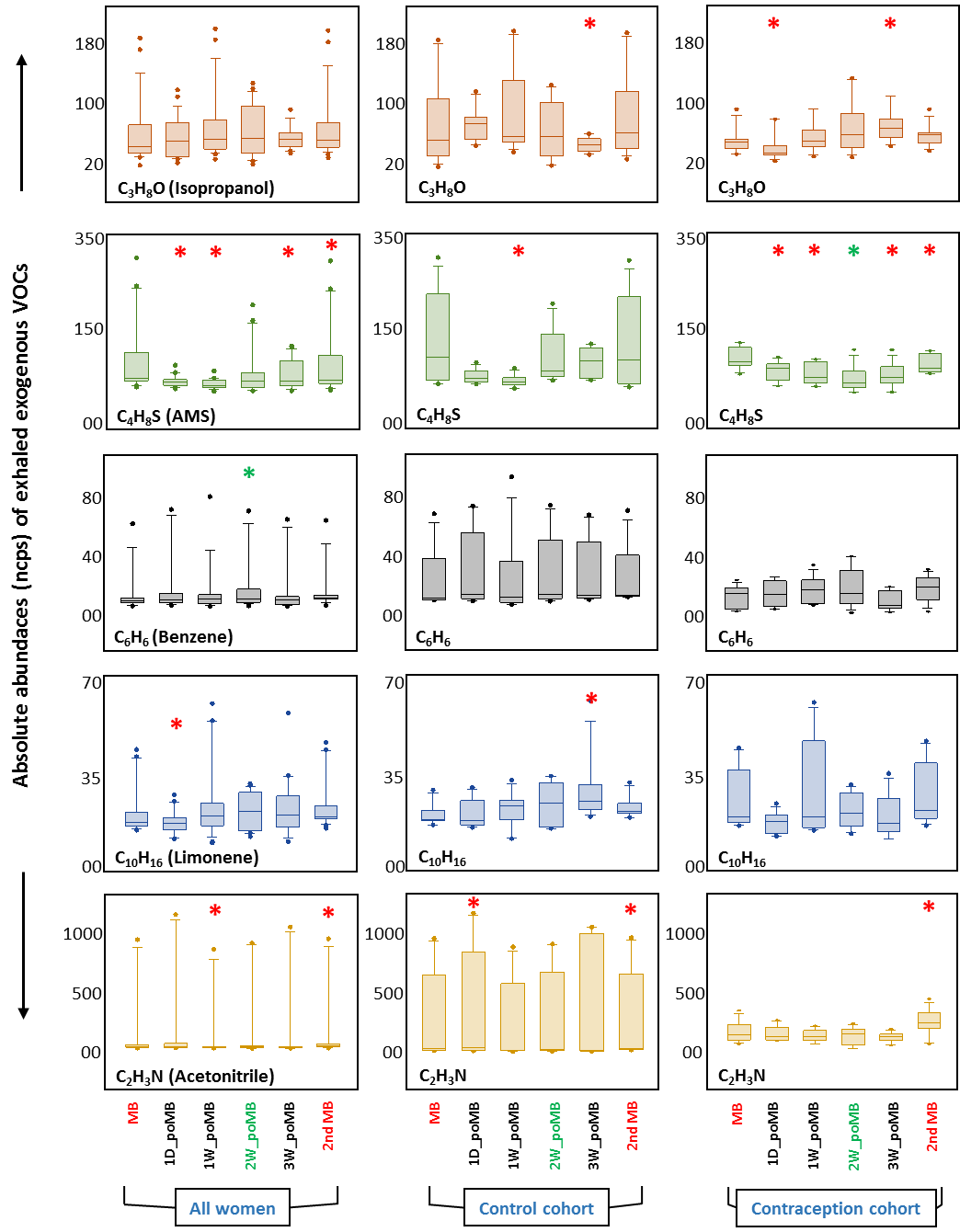


**Supplementary figure S1.** Statistical comparisons of four potentially exogenous VOCs from all women, control- and contraception cohort, respectively during different phases of their menstrual cycle. All Y-axis represent the median values of exhaled alveolar VOC abundances. All X-axis represent the six different measurement points (first menstrual bleeding to 2^nd^ menstrual bleeding phase) of the study. VOC data of all phases were compared to the corresponding median values in the initial ‘MB’ phase. The ‘*’ symbols (red and green) represent the statistically significant (i.e. *P*-value ≤ 0.005) differences in relation to the initial MB phase. A green ‘*’ is used to assign the ovulation phase.

| **All women** | | | | | | |
| --- | --- | --- | --- | --- | --- | --- |
| Protonated VOCs [g/mol] | Significance (*P ≤* 0.005)/ Median/60 s | Median Changes (%)/60 s | **Phases of menstrual cycle** | Median Changes (%)/60 s | Significance (*P ≤* 0.005)/ Median/60 s | Protonated VOCs [g/mol] |
| (Ammonia)H+ 18.03382 | ***N/A*** | ***0*** | ***MB*** | ***0*** | ***N/A*** | (Isopropanol)H+ 61.06479 |
|  |  |  |  |  |  |  |
|  | *0.004* | -2.31 | **1D-poMB** | 14.23 | *>0.005* |  |
|  |  |  |  |  |  |  |
|  | *>0.005* | 6.36 | **1W-poMB** | 18.80 | *>0.005* |  |
|  |  |  |  |  |  |  |
|  | *<0.001* | -9.85 | **2W-poMB** | 20.55 | *>0.005* |  |
|  |  |  |  |  |  |  |
|  | *>0.005* | 13.13 | **3W-poMB** | 18.76 | *>0.005* |  |
|  |  |  |  |  |  |  |
|  | *>0.005* | 0.78 | **2nd MB** | 16.58 | *>0.005* |  |
|  |  |  |  |  |  |  |
| (Acetone)H+ 59.04914 | ***N/A*** | ***0*** | ***MB*** | ***0*** | ***N/A*** | (AMS)H+ 89.04195 |
|  |  |  |  |  |  |  |
|  | *>0.005* | -3.42 | **1D-poMB** | -26.77 | *0.002* |  |
|  |  |  |  |  |  |  |
|  | *0.001* | 7.42 | **1W-poMB** | -56.65 | *0.001* |  |
|  |  |  |  |  |  |  |
|  | *<0.001* | -11.01 | **2W-poMB** | -23.34 | *>0.005* |  |
|  |  |  |  |  |  |  |
|  | *0.004* | 11.92 | **3W-poMB** | -24.98 | *0.004* |  |
|  |  |  |  |  |  |  |
|  | *>0.005* | -2.38 | **2nd MB** | -11.79 | *<0.001* |  |
|  |  |  |  |  |  |  |
| (Isoprene)H+ 69.06989 | ***N/A*** | ***0*** | ***MB*** | ***0*** | ***N/A*** | (Limonene)H+ 137.13248 |
|  |  |  |  |  |  |  |
|  | *>0.005* | -5.22 | **1D-poMB** | -5.19 | *>0.005* |  |
|  |  |  |  |  |  |  |
|  | *>0.005* | -18.10 | **1W-poMB** | 22.80 | *<0.001* |  |
|  |  |  |  |  |  |  |
|  | *<0.001* | -19.46 | **2W-poMB** | 38.08 | *>0.005* |  |
|  |  |  |  |  |  |  |
|  | *>0.005* | 1.33 | **3W-poMB** | 24.39 | *>0.005* |  |
|  |  |  |  |  |  |  |
|  | *>0.005* | -10.50 | **2nd MB** | 20.12 | *>0.005* |  |
|  |  |  |  |  |  |  |
| (DMS)H+ 63.0263 | ***N/A*** | ***0*** | ***MB*** | ***0*** | ***N/A*** | (Acetonitrile)H+ 42.03382 |
|  |  |  |  |  |  |  |
|  | *<0.001* | -48.85 | **1D-poMB** | -0.54 | *>0.005* |  |
|  |  |  |  |  |  |  |
|  | *0.005* | -64.47 | **1W-poMB** | -10.45 | *>0.005* |  |
|  |  |  |  |  |  |  |
|  | *<0.001* | -51.81 | **2W-poMB** | -6.34 | *>0.005* |  |
|  |  |  |  |  |  |  |
|  | *>0.005* | -46.83 | **3W-poMB** | -18.57 | *>0.005* |  |
|  |  |  |  |  |  |  |
|  | *0.002* | 24.47 | **2nd MB** | 21.06 | *<0.001* |  |
|  |  |  |  |  |  |  |
| (Benzene)H+ 79.05423 | ***N/A*** | ***0*** | ***MB*** | ***0*** | ***N/A*** | (Toluene)H+ 93.06989 |
|  |  |  |  |  |  |  |
|  | *>0.005* | 8.29 | **1D-poMB** | -8.38 | *>0.005* |  |
|  |  |  |  |  |  |  |
|  | *>0.005* | 14.71 | **1W-poMB** | -1.53 | *>0.005* |  |
|  |  |  |  |  |  |  |
|  | *0.002* | 19.11 | **2W-poMB** | 11.11 | *>0.005* |  |
|  |  |  |  |  |  |  |
|  | *>0.005* | 3.13 | **3W-poMB** | -14.89 | *>0.005* |  |
|  |  |  |  |  |  |  |
|  | *>0.005* | 26.14 | **2nd MB** | 15.18 | *>0.005* |  |
|  |  |  |  |  |  |  |
| (Hydrogen sulphide)H+ 34.995 | ***N/A*** | ***0*** | ***MB*** | ***0*** | ***N/A*** | (Methyl-propyl sulphide)H+ 42.03382 |
|  |  |  |  |  |  |  |
|  | *>0.005* | -8.44 | **1D-poMB** | -48.09 | *>0.005* |  |
|  |  |  |  |  |  |  |
|  | *>0.005* | 9.63 | **1W-poMB** | -17.65 | *>0.005* |  |
|  |  |  |  |  |  |  |
|  | *>0.005* | 38.98 | **2W-poMB** | -34.72 | *>0.005* |  |
|  |  |  |  |  |  |  |
|  | *>0.005* | -2.79 | **3W-poMB** | -17.15 | *>0.005* |  |
|  |  |  |  |  |  |  |
|  | *>0.005* | 40.72 | **2nd MB** | -21.53 | *>0.005* |  |
|  |  |  |  |  |  |  |

**Supplementary table S1:** Statistical significance of observed changes in exhaled end-tidal abundances of 12 different VOCs in all women. Absolute median values of VOC concentrations from the second minute (/60 s) of every measurement points were compared. **Median Changes (%)/60 s:** positive values represent an increase and negative values represent a decrease in absolute values. **Significance (*P*-value ≤ 0.005)/Median/60 s:** statistically significant differences between the reference value (from initial menstrual bleeding phase) and actual values from all other measurement points were assessed by means of repeated measurement-ANOVA on ranks.
